# The Influence of Thermal Properties Anisotropy on Subtractive Laser Processing of B_4_C/h-BN Composites

**DOI:** 10.3390/ma13225191

**Published:** 2020-11-17

**Authors:** Paweł Rutkowski, Karol Gala, Kamila Misiura, Jan Huebner

**Affiliations:** Department of Ceramics and Refractories, Faculty of Materials Science and Ceramics, AGH University of Science and Technology in Krakow, al. Mickiewicza 30, 30-059 Kraków, Poland; wattslaw.gala@gmail.com (K.G.); kamilamajamisiura@gmail.com (K.M.); huebnerj@agh.edu.pl (J.H.)

**Keywords:** boron carbide, hexagonal boron nitride, anisotropy, thermal properties, solid lubricate, laser processing, ceramic matrix composite

## Abstract

This work concerns boron carbide matrix composites with the addition of hexagonal boron nitride particles (h-BN) as a solid lubricate. The composite materials were hot-pressed and analysed in terms of phase, structure, and microstructure changes in relation to the h-BN content. The uniaxial pressure applied during the manufacturing process allowed the orientation of single h-BN particles and its agglomerates in perpendicular direction to the pressing axis. The anisotropy of heat transfer and thermal expansion coefficient (CTE) and density changes in relation to temperature are discussed. Thermal diffusivity and conductivity were measured in relation to the material direction by the laser flash analysis method (LFA). In this paper, understanding the heat flow and CTE changes allowed explaining the results of investigated subtractive laser processes of the manufactured composites. The laser ablation process was conducted on B_4_C/h-BN composites in parallel and perpendicular direction to each other. It was done in a continuous work (CW) mode at 50 W with a 40 µm spot and 3 mm/s beam travel speed. The influence of h-BN particles and their orientation on thermal properties is discussed. The effect of laser processing on B_4_C/h-BN composites was also discussed in relation to the material surface roughness measured with a confocal microscope, microstructure observations, density, and thermal properties changes in relation to the material direction.

## 1. Introduction

The boron carbide is a low density material of 2.51 g/cm^3^ with rhombohedral crystallographic structure including a high melting point of about 2400 °C, very high hardness of 38 GPa, a good elastic, excellent wear and mechanical properties, and high neutron absorption cross section [1,2,3,4]. Due to these properties, it is widely used as sand blasting nozzles, armor plates, sliding rings, neutron-shielding materials in nuclear industry, milling agents, grinding material, hard coatings, and also boron neutron capture therapy (BNCT) [5,6,7,8,9].

The hexagonal boron nitride, also called white graphite, was very often investigated by researchers due to the possibilities to decrease the friction coefficient for cutting tools and bearing sliding applications [10,11]. This phase has been inserted into various oxides, nitrides, and carbides of advanced ceramic materials, for example: Si_3_N_4_, SiC, Al_2_O_3_, B_4_C, SiAlON.

The first works concerning anisotropic properties of B_4_C-hBN ceramics were made by Robert Ruh in 1992 for 0, 20, 40, 60, 80, and 100 vol.% of hexagonal boron nitride. He noticed that there was a large anisotropy of thermal properties [12,13]. Even in the case of the hot-pressing process, a higher concentration of hBN will increase the material porosity and decrease the mechanical properties, which was confirmed by Li et al. They noticed that a 20% h-BN addition will improve the friction coefficient of such composites and simultaneously, there is an increase in the wear coefficient [10,14,15,16,17,18,19,20] when 5 wt % of h-BN in the material is exceeded. They found that in distilled water conditions, the wear behavior of B_4_C/h-BN composites against steel is improved. In dry conditions, limiting the addition of h-BN keeps a good tribological performance of about 10%. In the research made by Ruh, there is a lack of composition between 0 and 20 and 40 vol.%, therefore, for the h-BN content, it is possible to keep a balance between the mechanical properties and thermal conductivity. The author of this paper investigated thermally and mechanically boron carbide 2–8 wt % of h-BN composites, which were hot-pressed with a chromium carbide sintering aid [21]. In the paper, the addition of h-BN did not decrease the thermal properties significantly, but instead, allowed keeping good values of mechanical properties at a similar or slightly higher level to the reference sample. The introduced 2D phase led to a decrease in the friction coefficient and wear rate.

Based on the works by Ruh and previous research made by the authors of this paper, further research in the B_4_C/h-BN system was made and presented. For 0.5, 1, 2, 4, 8, 16, and 32 vol.% of the h-BN content the following anisotropic properties were examined in this paper: Linear thermal expansion coefficient, diffusivity, and thermal conductivity. The obtained thermal data were used to discuss an influence of the added 2D phase on the material cut shape quality after the laser ablation process under a CW mode laser processing. The correlation between thermal properties and laser ablation of boron carbide/h-BN composites provides new information crucial for understanding the rapid heat processing of this composite system and gives new data in the literature concerning the material behavior during laser processing.

## 2. Preparation and Examination Route

To manufacture the boron carbide–hexagonal boron nitride composites, the following commercially available powders were used: Boron carbide grade HS nr AB134566 (97% purity, 2.51 g/cm^3^ density, and 0.8 µm average grain size) from the H.C. Starck company (Karlsruhe, Germany) and BO-501 (99.9% purity, 2.25 g/cm^3^ density, and 0.3–0.7 µm grain size) from the Atlantic Equipment Engineers company (Bergenfield, USA). No additional sintering agent was used. The powders were mixed together to obtain mixtures containing 0, 0.5, 1, 2, 4, 8, 16, 32 vol.% of h-BN phase, which fulfilled the gaps in Ruh mixtures’ compositions and further investigations.

**Figure 1:**
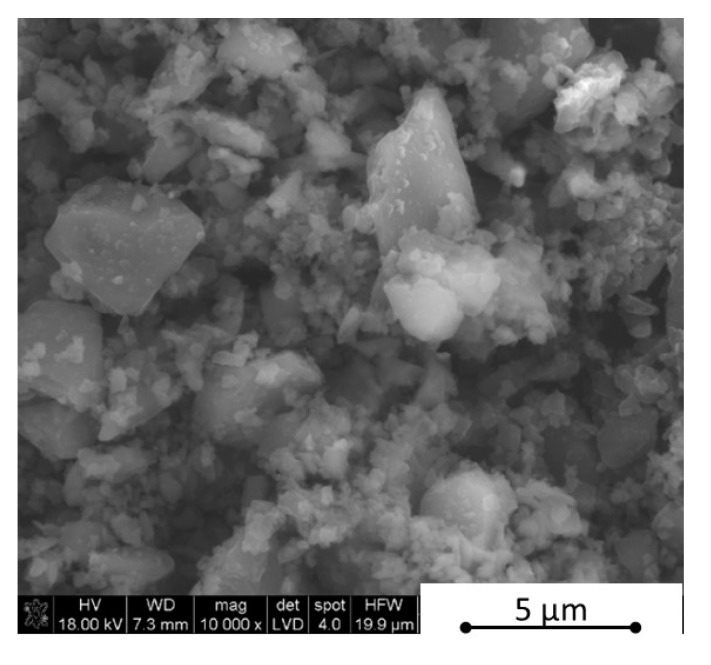
Morphology of commercial boron carbide grade HS of H.C. Starck.

**Figure 2:**
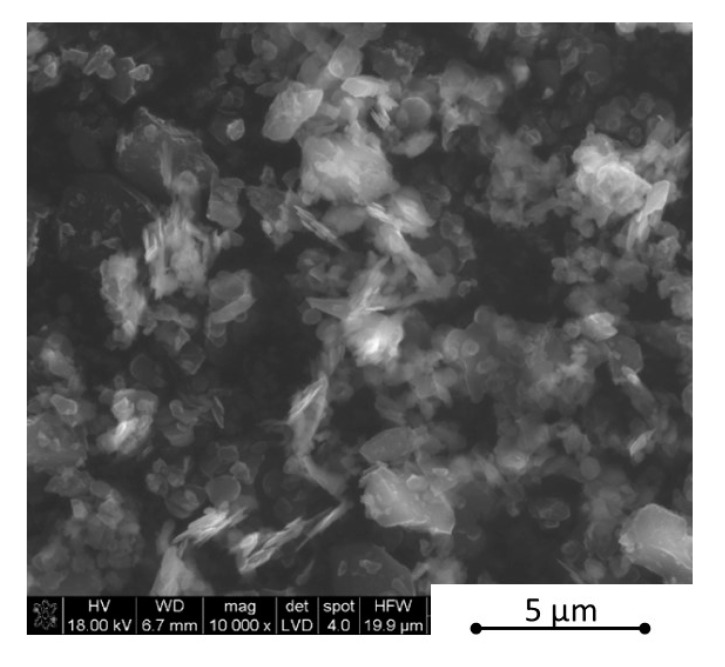
Morphology of the B_4_C—32 vol.% hexagonal boron nitride particles (h-BN) mixture.

As the first step, the boron nitride powder was de-agglomerated in an ultrasonic bath in isopropyl alcohol for 4 h. During this step, boron carbide was added and the ultrasonic was used for 2 h in order to get a uniform powder mixture. Afterwards, the composition sets were homogenized for 20 h in a laboratory rotational dissolver, modelTD100 (Pendraulik-Teja) (Springe, Germany) with 900 rpm/min. Such prepared mixtures were dried using a magnetic stirrer with a heating up mode. This step took up to 3 h with 200 rpm/min. The phase compositions were checked by the XRD analysis (PANalitycal X-Ray Diffractor with X-Pert HighScore software) (Almelo, The Netherlands) and morphology by SEM observations (FEI Nova Nano SEM) (Brno, Czech Republic). The morphology examples of the powders are shown in Figure 1 and Figure 2 and the powder compositions are collected in Table 1. After the de-agglomeration of initial powders, as a result of the powder mixtures homogenization step, we observed two B4C-I and B4C-II boron carbide phases and also two h-BN-I and h-BN-II hexagonal boron nitride phases, which showed slightly different lattice parameters.

The prepared granulated mixtures were hot-pressed using a Thermal Technology LLC HP apparatus (Santa Rosa, CA, USA) at 2150 °C under 25 MPa for 30 min in argon flow. The heating rate was 10 °C/min and the 25 mm diameter samples with 8 mm thickness were obtained.

The apparent density of hot pressed composites was measured by the hydrostatic method. The relative density was calculated and the average XRD phase composition analysis was taken for this calculation. Measurements were made for three samples of each composition. Sinters were polished in different material directions using diamond and silica media. The phase composition was investigated by XRD analysis on the sample cross-section and on its surface in perpendicular to the pressing direction. SEM observations were made on the samples’ surfaces in parallel and perpendicular direction to the pressing axis, as shown on the example of laser treatment investigations. The element distribution analysis was carried in order to identify phases in the material’s microstructure.

Thermal diffusivity and thermal conductivity measurements were carried out using the laser flash analysis method on the Netzsch LFA apparatus (Selb, Germany). The material was investigated at 25 °C, 50 °C, 100 °C, 300 °C, 600 °C, and 900 °C in argon flow. The specific heat was calculated on the base of the thermodynamic data available in the literature [22].

The confocal microscope Olympus Lext OLS4000 (Tokyo, Japan) was used to establish the material surface roughness (R_a_), which can have an influence on laser beam energy absorption and laser treatment. The examined materials were then taken under laser subtractive processing using the JKLaser 5000 (Rugby, UK) ytterbium doped fiber laser with a 1064 nm wavelength with a maximum power of 200 W (JK200FL). The trials were made under the argon flow coming out from the standard cutting head nozzle, where the argon pressure was set to 2 bars. The continuous wave (CW) mode with a 50 W power laser beam was used to shape the composite material. The laser beam spot was 40 microns. The process speed was set to 3 mm/s, with a single laser beam pass. The reference and composite material was processed in parallel and perpendicular direction to the material pressing axis of hot-pressing. After the process, the laser treated material was investigated on the cross-section of the laser cut surface by scanning electron microscopy (SEM).

## 3. Results and Discussion

### 3.1. Densification and Phase Analysis

**Figure 3:**
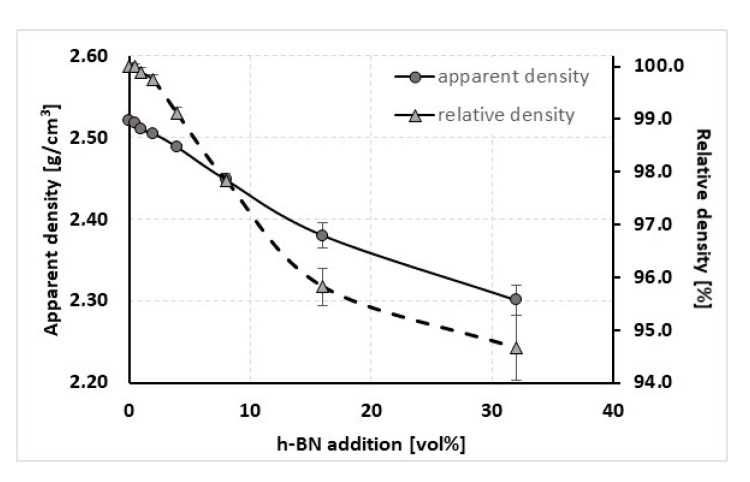
Densification of B_4_C/h-BN composites.

**Figure 4:**
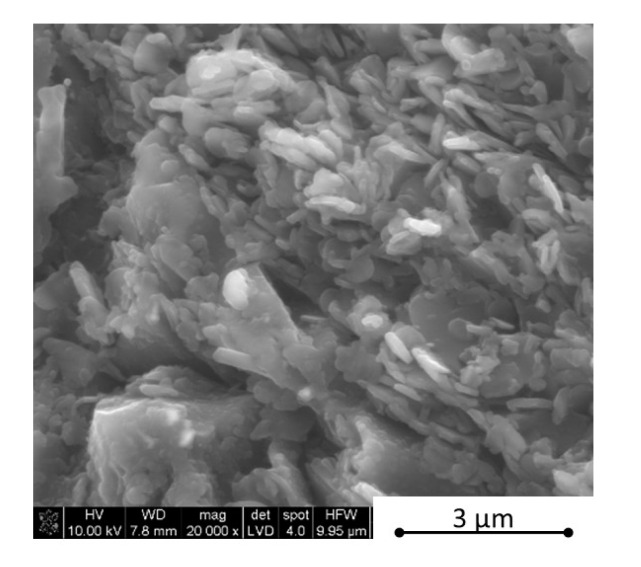
Fracture SEM observation of B_4_C/16 vol.% h-BN composites.

The apparent density results measured by the hydrostatic method is presented in Figure 3. The obtained data shows that dense materials can be obtained only with the addition of up to 8 vol.% of hexagonal boron nitride, which is higher than the literature data [13]. A further increase of h-BN content led to a decrease in the material density, which results from porous agglomerates of this phase (Figure 4). The density measurement error increases with the h-BN content, which is connected with the h-BN distribution, water adsorption to agglomerates, and a possible absence of h-BN after the HP graphite foil removal. The existence of hexagonal boron nitride agglomerates can lead to a decrease of the material friction coefficient [21,23]. Such agglomerates can strongly adsorb a large amount of water from the atmosphere, which was visible on the dilatometric curve changes as a negative jump on the curve. Such material required an additional time in the vacuum chamber in order to remove the adsorbed water before the final CTE analysis, which is described in a further part of this paper.

**Figure 5:**
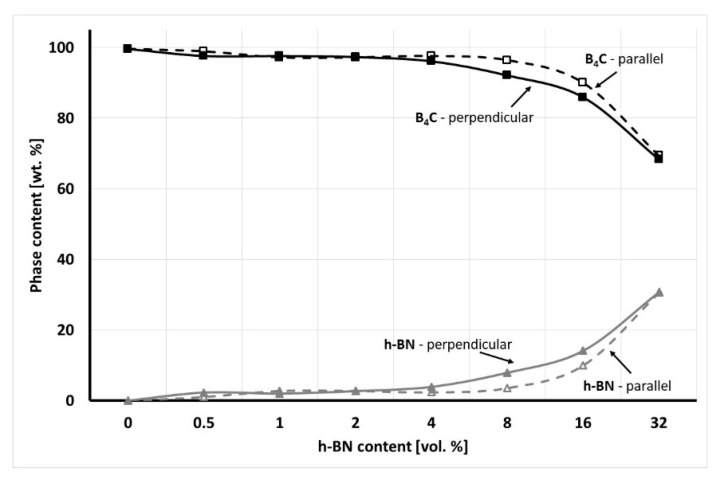
Phase composition analysis versus the B_4_C/h-BN composite direction.

Due to the uniaxial pressure applied during the hot-pressing process, there should be a difference in the XRD/Rietveld analysis measured in various material directions. XRD peak intensities of selected material phases show various values in different material directions, which have a translation into their content. The result of the phase composition analysis made in perpendicular (in-plane) and parallel (out-of-plane) direction to the pressing axis is presented in Figure 5.

**Figure 6:**
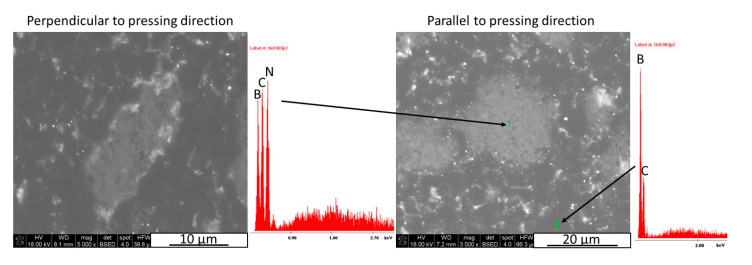
SEM/EDS analysis and phase identification on an example of 16 vol.% h-BN composite microstructure.

The XRD/Rietveld analysis showed that for the hot-pressed material with up to 2 vol.% there is almost no difference in the composite phase composition in relation to the material direction. The change of the selected phase concentration was noticeable for composites with 8–16 vol.% of hexagonal boron nitride due to the 2D phase orientation in the material as a result of applied pressure. For the highest content of h-BN solid lubricant, there was almost no difference due to the high content of even oriented h-BN agglomerates. In the case of a perpendicular direction, 1% of B_2_O_3_ was present in the samples with 32 vol.% of h-BN. It formed due to the oxidation of h-BN agglomerates. Some of the boron oxide can be present on the initial B_4_C powders grain surface, which cannot be detected due to its low amount. It can happen, for example, during the cutting or polishing process of the sample for XRD analysis. Also small quantity of tungsten borides were found with the used analysis detection level, about 0.4% in pure material and 1 vol.% h-BN composite. The W_x_B_y_ tungsten boride is a result of the reaction between boron carbide and tungsten carbide impurities. The identification of the phase in the material microstructure was made by the SEM/EDS analysis and presented in Figure 6. The EDS analysis was made on the h-BN agglomerate in order to get a good element count. The dark grey colour belongs to boron carbide and the light grey to hexagonal boron nitride.

### 3.2. Microstructure

**Figure 7:**
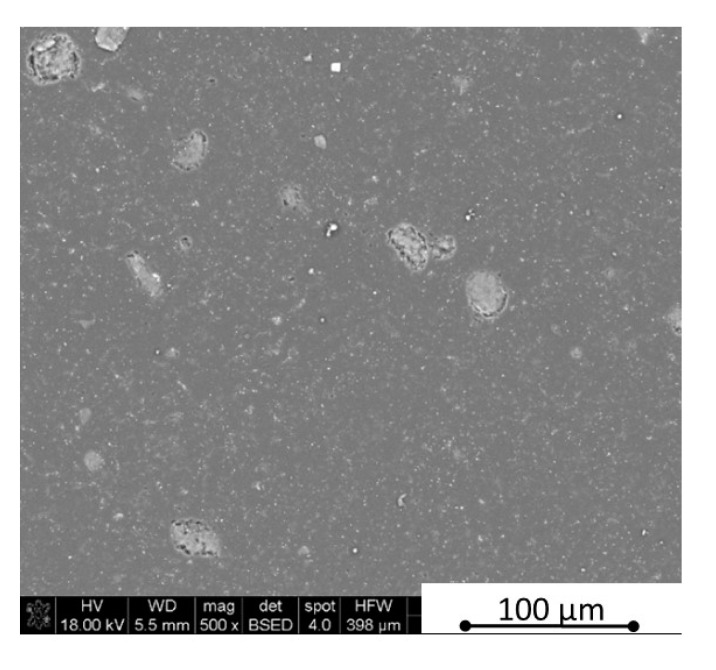
SEM surface observations of composites with 4 vol.% h-BN (in parallel direction).

**Figure 8:**
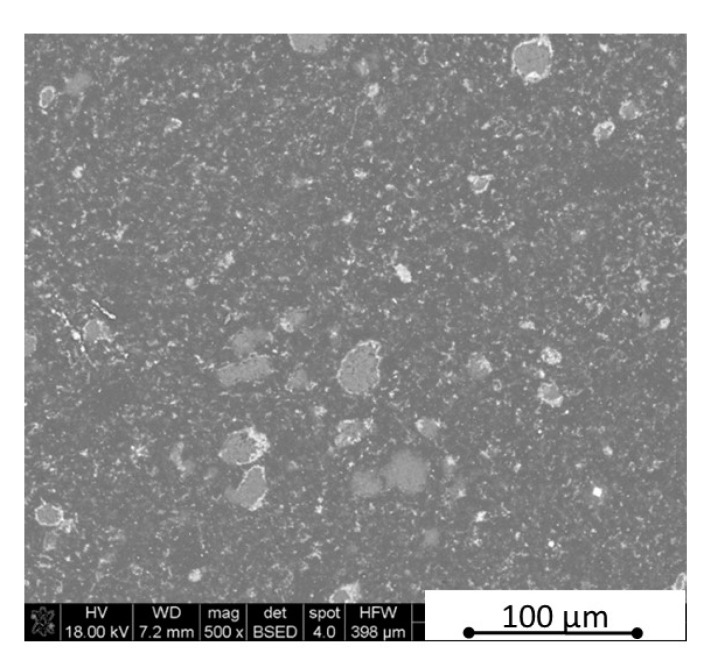
SEM surface observations of composites with 16 vol.% h-BN (in parallel direction).

**Figure 9:**
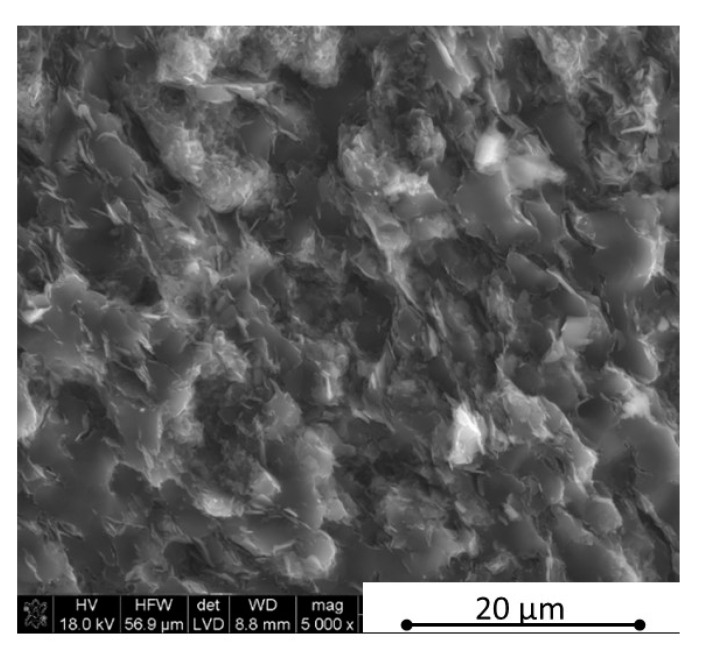
SEM fracture observations of composites with 16 vol.% h-BN.

**Figure 10:**
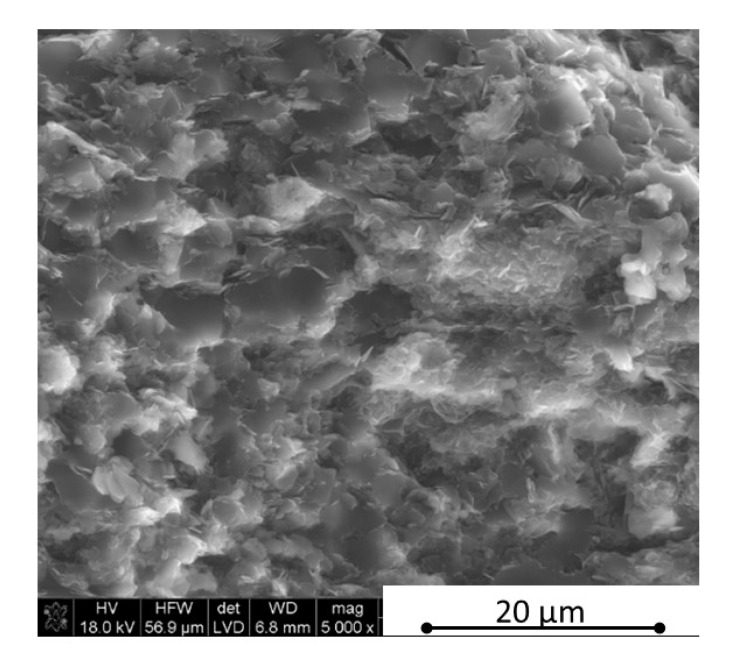
SEM fracture observations of composites with 32 vol.% h-BN.

The microstructure observations were made using scanning electron microscopy in parallel and perpendicular direction to the pressing axis of the HP process. The SEM polished surface observations made in parallel direction to the pressing axis are presented in Figure 7 and Figure 8. The observation of reference material indicates the existence of an additional “white” phase in the microstructure, which was also noticed in the B_4_C/h-BN composites and can come from small WC impurities in the initial powders’ mixtures. The performed XRD phase composition and EDS element distribution analysis confirmed a formation of tungsten borides as a result of the tungsten carbide reaction with a material matrix during the sintering process. Tungsten carbide was found in up to 0.1% concentration in the material initial mixtures. The tungsten boride was a contamination in the quantity of up to 0.4% in sinters. The images in Figure 7 and Figure 8 show an existence of up to 20 µm size agglomerates of hexagonal boron nitride for samples in the pressing axis direction. The content of agglomerates increases with the rising concentration of the introduced h-BN to the composites. The fracture images of composites with 16 and 32 vol.% of h-BN presented in Figure 9 and Figure 10 also confirm some fine h-BN particles/flakes, which are well distributed in the B_4_C matrix and oriented in one direction in the matrix (Figure 10).

**Figure 11:**
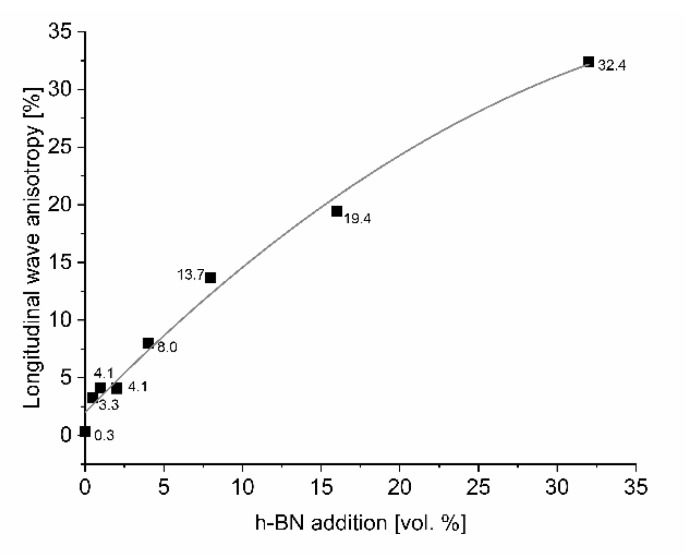
Anisotropy of longitudinal wave velocity of B_4_C/h-BN composites.

In order to confirm the anisotropic character of manufactured composites, the ultrasonic measurements of longitudinal wave velocity in relation to the material direction were done. The results are shown in Figure 11. The ultrasonic results show the anisotropy of wave velocity reaching 32% for 32 vol.% of h-BN. It can also result from the porosity of h-BN agglomerates, where the shape of pores has an important role.

### 3.3. Thermal Properties and Thermal Stability

**Figure 12:**
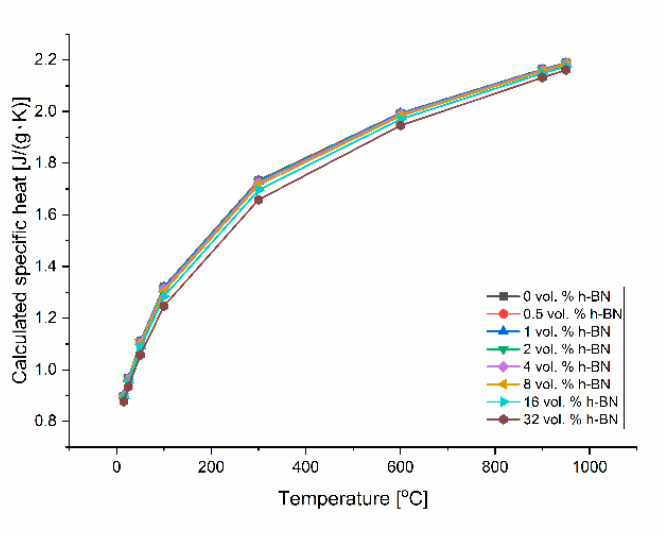
Calculated specific heat versus temperature on the base of NIST database [22].

**Figure 13:**
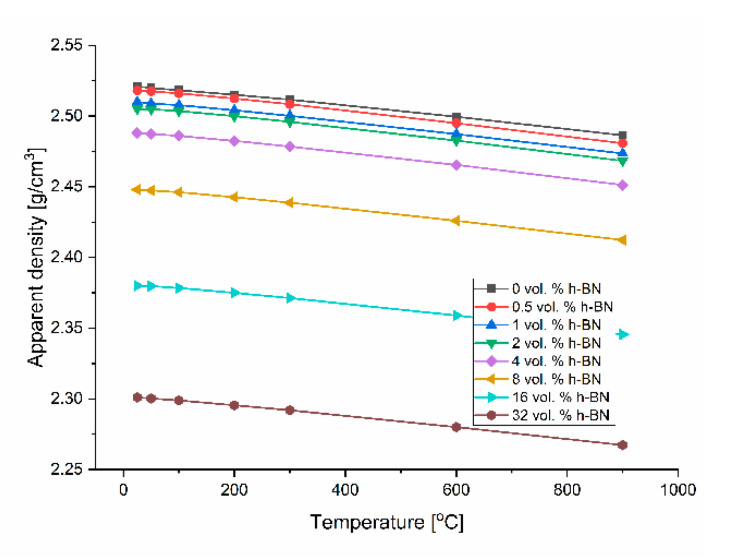
Experimental density changes versus temperature.

**Figure 14:**
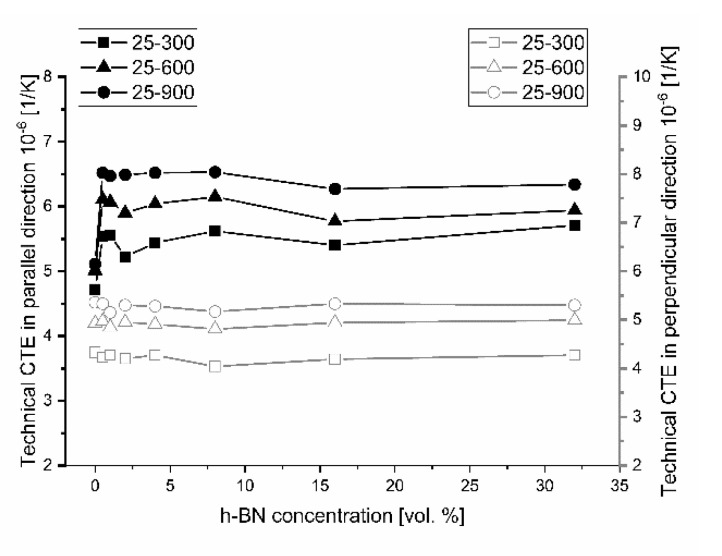
Material technical thermal expansion coefficient versus material direction. Empty marks are for the perpendicular direction to the pressing axis (in two-dimensional (2D) plane), and filled marks are for the pressing direction (out-of-plane).

**Figure 15:**
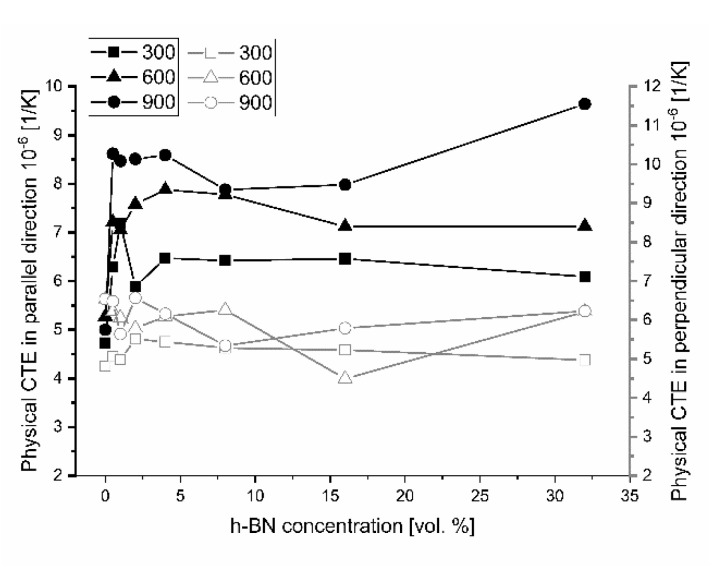
Material physical thermal expansion coefficient versus material direction. Empty marks are for the perpendicular direction to the pressing axis (in 2D plane), and filled marks are for the pressing direction (out-of-plane).

The determination of thermal conductivity by the direct thermal diffusivity analysis on a LFA apparatus cannot be made without specific heat data, linear material dimension changes, and density changes by dilatometry. The calculated specific heat on the base of thermodynamic data [22] and the calculated density on the base of dilatometric measurements are shown in Figure 12 and Figure 13. The density changes were calculated on the base of the thermal expansion coefficient measured in various material directions, which is presented in Figure 14 for the technical linear CTE and in Figure 15 for the physical linear CTE. The conducted measurements and calculations showed a slight decrease of material density with the increased temperature, which has a negligible influence on the thermal conductivity analysis. For future purposes, the mathematical functions were fitted to the experimental data and collected in Table 2. Figure 14 shows a similar behavior of density changes for all of the manufactured polycrystalline materials.

**Figure 16:**
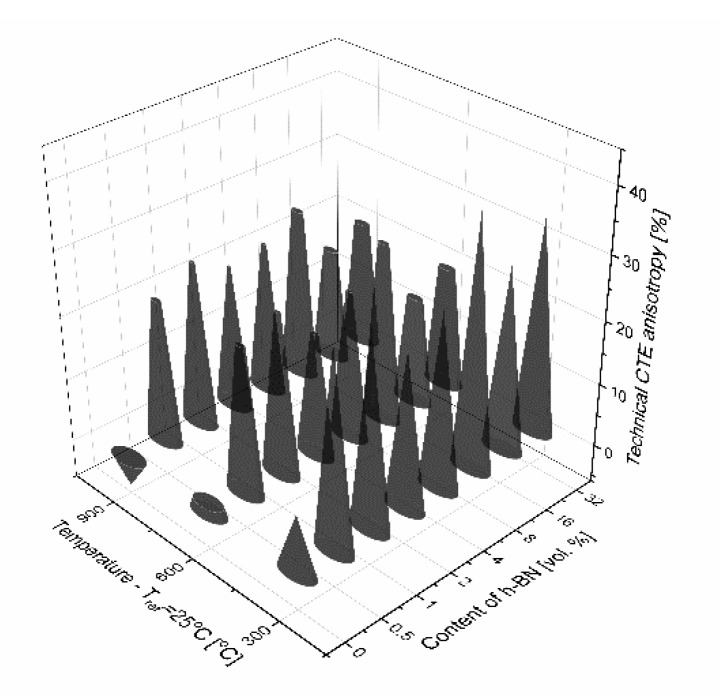
Anisotropy of the technical thermal expansion coefficient.

**Figure 17:**
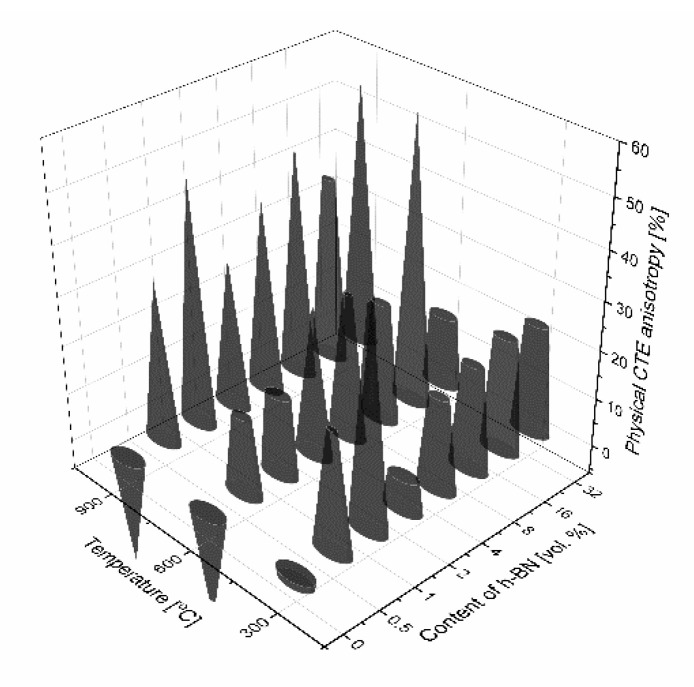
Anisotropy of the physical thermal expansion coefficient.

The results of thermal expansion coefficient shown in Figure 14 and Figure 15 indicate high anisotropy material linear changes versus material direction. A sudden increase of the technical and physical CTE value in the perpendicular direction begins for 0.5 vol.% of the h-BN content in the composite. A higher addition of h-BN does not significantly change the technical CTE. For the parallel direction to the pressing axis, it stays on the level of 5.4 × 10^−6^ 1/K for 25–300 °C measurement range, 5.9 × 10^−6^ 1/K for 25–600 °C measurement range, and 6.3 × 10^−6^ 1/K for 25–900 °C measurement range. For the perpendicular direction to the pressing axis, it is as follows: 4.2 × 10^−6^ 1/K for 25–300 °C measurement range, 4.9 × 10^−6^ 1/K for 25–600 °C measurement range, and 5.3 × 10^−6^ 1/K for 25–900 °C measurement range. The dilatometric analysis showed the material linear changes fluctuation in the case of physical CTE, which varied in relation to the h-BN content and especially the high temperatures visible for 32 vol.% h-BN at 900 °C. The calculated data of the technical and physical CTE anisotropy are illustrated in Figure 16 and Figure 17. For high temperatures, the physical CTE anisotropy stays on the level of 50%. For lower temperatures, it is mostly between 20% and 30% (Figure 17). For the technical CTE, all the measured temperature ranges are between 20% and 30% (Figure 16).

**Figure 18:**
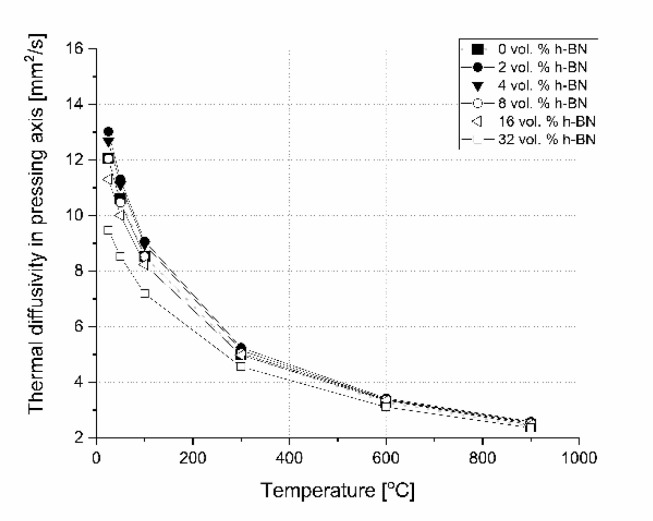
Thermal diffusivity versus temperature and h-BN content in the pressing direction.

**Figure 19:**
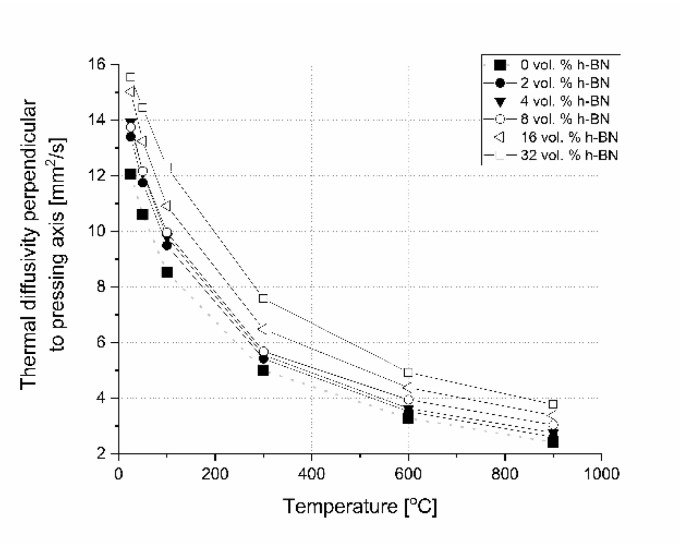
Thermal diffusivity versus temperature and h-BN content perpendicularly to the pressing direction.

In the literature, there is no excessive information concerning the influence of 0–32 vol.% h-BN and the applied pressure of the hot-pressing process on the anisotropy of heat transport versus temperature in the boron carbide composite matrix. This is why the examples of thermal diffusivity data measured as a function of temperature and material direction are presented in Figure 18 and Figure 19. For lower contents of h-BN, there is a slight increase of heat transfer due to the high material densification above 99%, low content of flat defects (interphase boundary), and second 2D high conductive phase and low material anisotropy (Figure 11). A similar situation occurs when using a low quantity of graphene powder (GNP), where a small amount of 2D particles leads to an increase of thermal conductivity [23]. In the case of parallel direction to the pressing axis (Figure 18), at lower temperatures diffusivity decreases with the h-BN addition higher than 4 vol.%. It is caused by a higher material porosity connected mostly with h-BN agglomerates, increasing the concentration of flat defects (fractures in Figure 9 and Figure 10) and orientation of 2D particles. The thermal conductivity of a platelet shaped h-BN is above 200 W/(m∙K) in the plane direction and around 2 W/(m∙K) through the plane direction [24]. The authors of [24] confirmed that in the plane orientation of h-BN, larger thermal properties than in the perpendicular direction make the 2D particle plane. They also showed that very small particles and large agglomerates tend to orient less, therefore, it can also have an influence on thermal properties, which is its value in our case in perpendicular direction to the pressing axis. The scattering of phonon at boundaries of the studied samples will cause the heat transfer to decrease mainly in perpendicular direction to the pressing axis due to the material anisotropy. For higher temperatures (900 °C) all curves reach values between 2.3 and 2.6 mm^2^/s. The results of thermal diffusivity measured in perpendicular direction show higher values for the higher h-BN content in a whole range of temperatures. This parameter decreased with the temperature more slightly than in the parallel direction. At 900 °C, increasing the h-BN content thermal diffusivity changes from 2.4 to 3.8 mm^2^/s is visible in Figure 19.

**Figure 20:**
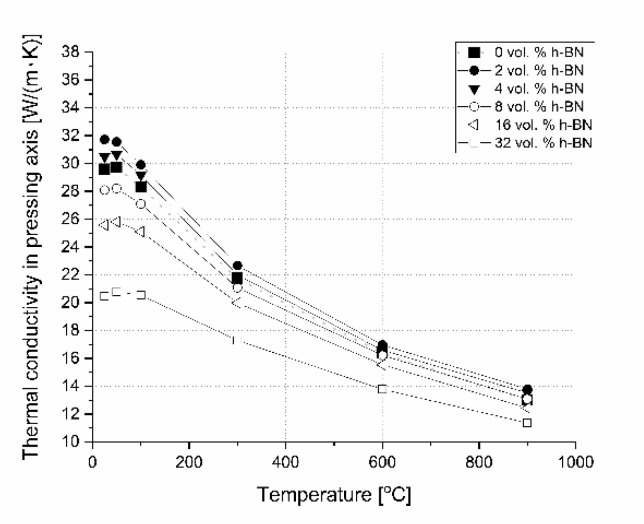
Thermal conductivity versus temperature and h-BN content in pressing direction.

**Figure 21:**
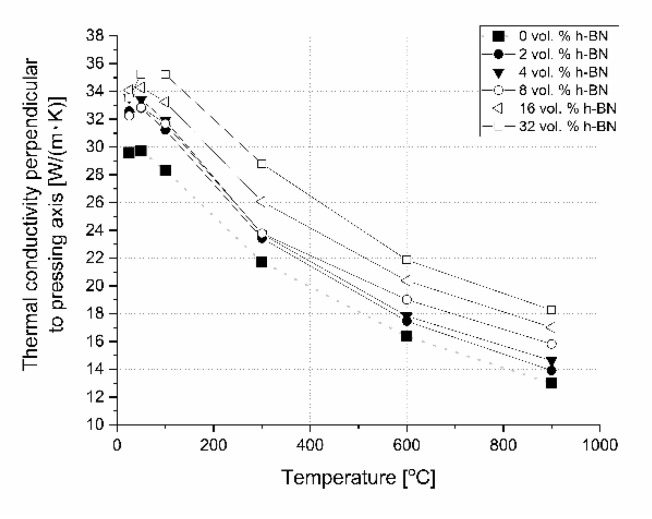
Thermal conductivity versus temperature and h-BN content perpendicularly to the pressing direction.

Concerning the pure nonstoichiometric boron carbide, the electronic transport causing polarons and phonon a play role in thermal conductivity and it was explained in NASA papers [25]. In our paper, we describe the B_4_C/h-BN composites thermal conductivity calculated on the base of thermal diffusivity, material density, and specific heat changes. Results of this parameter are illustrated in Figure 20 for the parallel measurement direction to the pressing axis (out-of-plane) and in Figure 21 for the perpendicular measurement direction to the pressing axis (in-plane). Due to specific heat in most of the manufactured composites, thermal conductivity increases slightly in the 25–50 °C range. The increase of thermal conductivity is caused by high conductive 2D particles, high material densification, and lower 2D agglomerates content. For higher temperatures, there is a decrease of this property possibly due to phonon-phonon scattering (Figure 20 and Figure 21). The addition of a low amount of h-BN allows obtaining slightly about 7% higher thermal properties in parallel direction to the pressing axis than for the reference sample of 29.6 W/(m·K). In the pressing direction, the higher decrease in thermal properties of this material with a temperature rise is also visible for composites with a higher h-BN content above 8 vol.%, which is marked by empty chart markers (Figure 20). Here, we have additional phonon scattering at boundaries and higher material porosity, which influence the lower heat transfer especially in the pressing direction. The thermal conductivity at 900 °C in this material direction is in the level of 11.3–13.8 W/(m·K)—the highest value is for 2 vol.% h-BN addition and the lowest for 32 vol.% of this phase. Due to the high material densities (Figure 3), in the case of low h-BN concentrations the decrease of thermal properties in low temperatures can be explained by the finer microstructure following the h-BN addition, therefore, increasing flat defects (usually 2D particles are limiting the grain growth of matrix during the hot-pressing process). The fine material microstructure is presented in Figure 9 and Figure 10. For higher temperatures, the phonon-phonon scattering and phase orientation mostly influence thermal conductivity. In thermal conductivity, the thermal barrier existing on the B_4_C/h-BN interface is very important, which was noticed by Ruh [13]. He stated that the mismatch in the elastic modulus of B_4_C and h-BN phases will cause phonon scattering. He also discussed that the microcracking or interracial adhesion can form a thermal barrier—which is in our case during the 2D particle orientation. Moreover, he added that Niihara discussed a possible thermal barrier on an example of Si_3_N_4_-BN composites. In our case, the higher amount of h-BN gives significantly lower thermal properties values, which is not only connected with an h-BN single particles/agglomerates orientation, but also higher porosity (lower density—Figure 3) existing in h-BN agglomerates. In the case of perpendicular direction, thermal conductivity values increase with the addition of a h-BN solid lubricant, which is visible for low temperatures up to 100 °C (Figure 21), but for higher temperatures they stay much higher than the reference sample. It can be explained by the h-BN particle orientation in perpendicular direction to the pressing axis (also confirmed by ultrasonic measurements) and agglomerates that the shape is elongated in this direction. The literature says that removing agglomerates or limiting their size can give a higher anisotropy, resulting in larger values of thermal properties in the 2D particle orientation [24], which can be a task for future research. In this direction, thermal conductivities at 900 °C stay above 13 W/(m K) reaching 18.3 W/(m·K) for the case of the highest h-BN concentration. The decrease of thermal properties in perpendicular direction in the temperature function is less aggressive than in the parallel case. For a future investigation, the fitted mathematical functions of thermal conductivity are given in Table 3.

**Figure 22:**
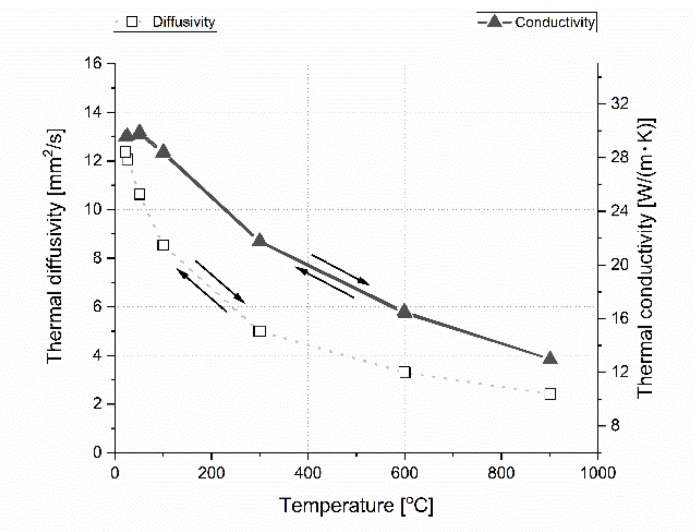
Stability investigation of thermal diffusivity and conductivity of B_4_C reference material in parallel direction. The arrows indicate the direction of heating and cooling during the measurement.

**Figure 23:**
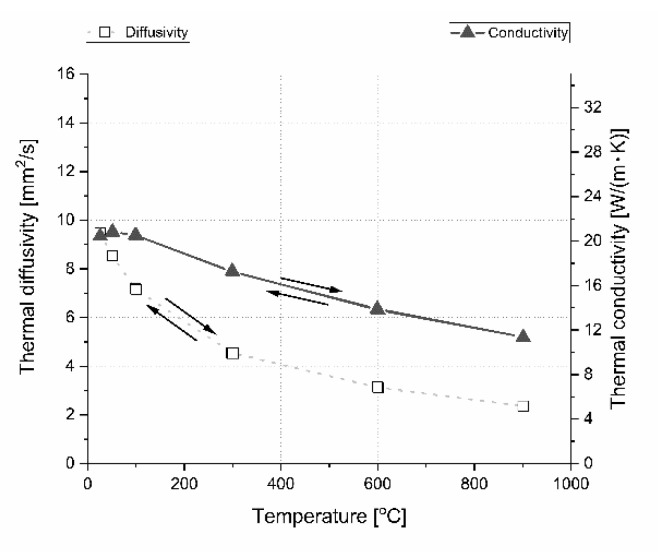
Stability investigation of thermal diffusivity and conductivity of B_4_C with 32 vol.% h-BN in parallel direction. The arrows indicate the direction of heating and cooling during the measurement.

The material properties can change as a result of: Oxygen contamination in argon protective gas, high temperature, long material treatment, and laser beam processing during the LFA measurement. This is the reason why two extreme sample compositions of the reference boron carbide polycrystalline material and one with 32 vol.% h-BN were taken under thermal conductivity measurements during heating and cooling steps. Results of this cyclic experiment are presented in Figure 22 and Figure 23. The obtained results show that there is almost no influence of laser processing, temperature, and gas purity on the thermal properties of boron carbide based polycrystalline materials with and without the h-BN dispersed phase. The material is thermally stable in a protective atmosphere.

**Figure 24:**
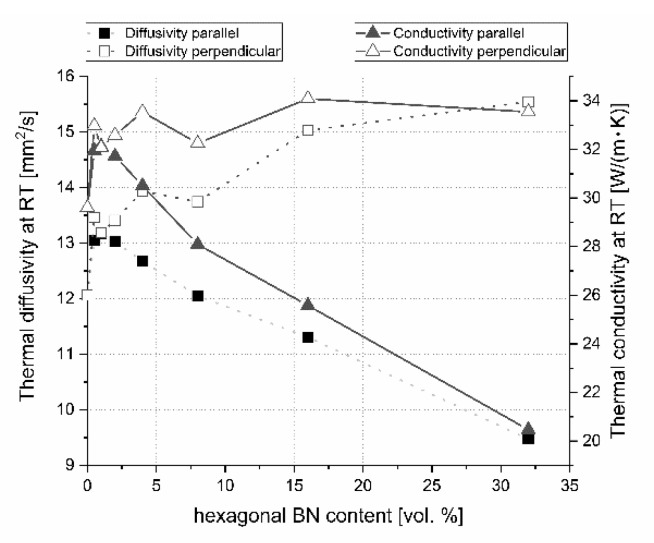
Thermal diffusivity and conductivity changes versus material direction and boron nitride content at room temperature (RT).

**Figure 25:**
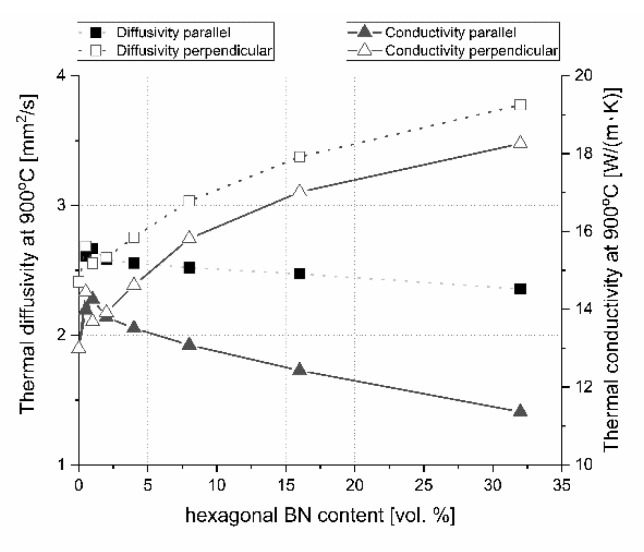
Thermal diffusivity and conductivity changes versus material direction and boron nitride content at 900 °C.

For the onset of laser processing description, the heat transfer properties at room temperature are very important (Figure 24). During the process, material properties at higher temperatures become much more interesting. The change of thermal diffusivity and conductivity at 900 °C is illustrated in Figure 25, which is the approximate temperature that stays around the laser-treated place for a short time after the interaction between the laser and sample.

The addition of hexagonal boron nitride will lead to an anisotropy of the thermal conductivity/diffusivity as a result of the dispersed particles and pores orientation in the material microstructure. For room temperature (RT) conditions in the pressing axis direction, an increase of h-BN particles will cause a strong decrease in thermal properties especially in the case of its higher h-BN content. A different situation occurs in the perpendicular direction where the heat transfer is faster. A similar situation can be met in high temperatures. There is a large thermal properties anisotropy at low and high temperature which increased with the higher h-BN content in the composites, as clearly visible in Figure 24 and Figure 25. This situation can have an influence on the material treatment, which is presented in the next part of this paper.

### 3.4. Subtractive Laser Processing

**Figure 26:**
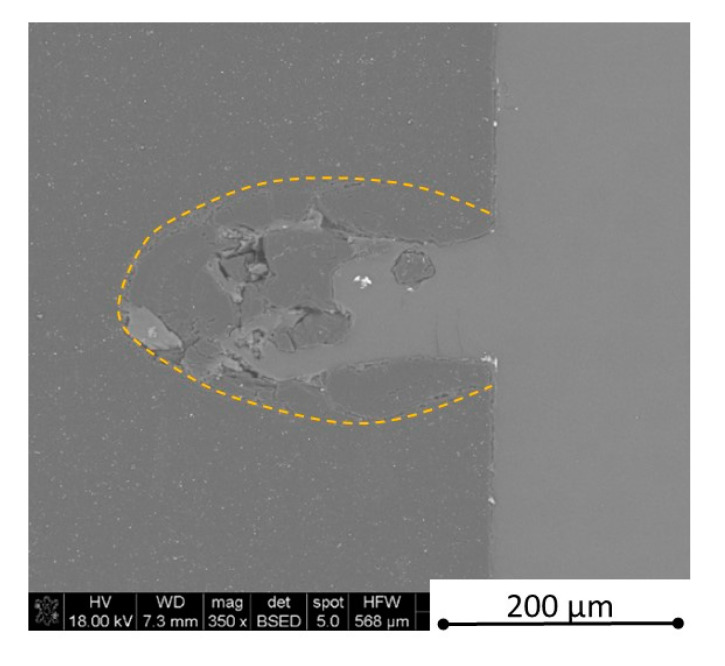
SEM observations of a 50 W laser processed B_4_C reference sample (high surface roughness).

**Figure 27:**
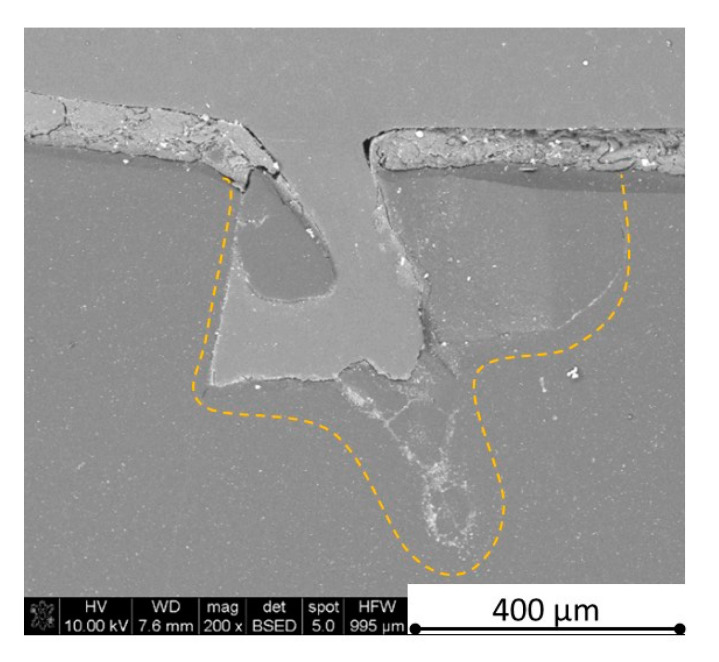
SEM observations of a 50 W laser processed B_4_C reference sample (high surface roughness).

**Figure 28:**
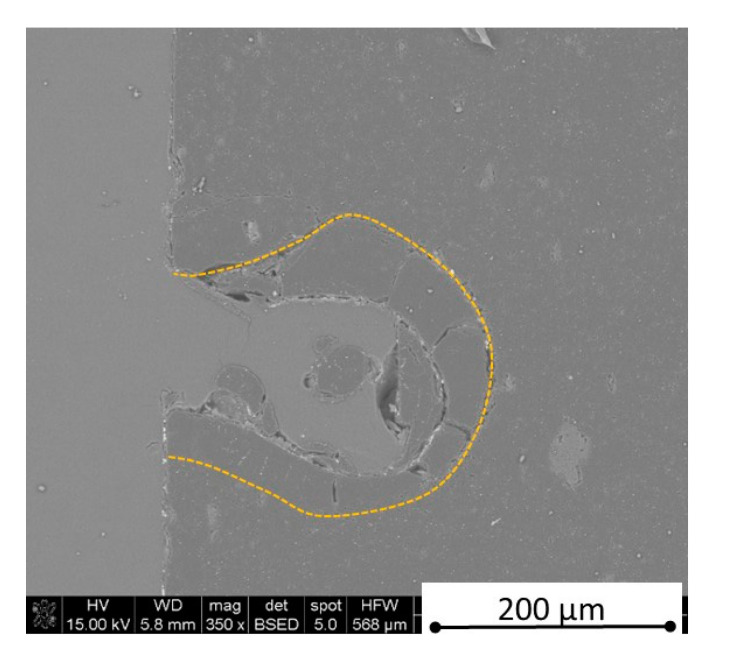
SEM observations of a 50 W laser processed B_4_C—4 vol.% h-BN in pressing direction (high surface roughness).

**Figure 29:**
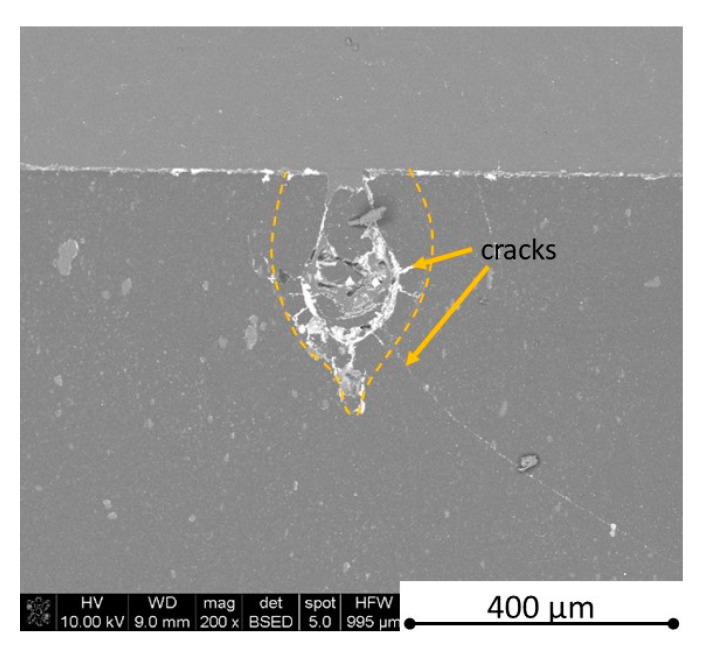
SEM observations of a 50 W laser processed B_4_C—4 vol.% h-BN in perpendicular direction (high surface roughness).

**Figure 30:**
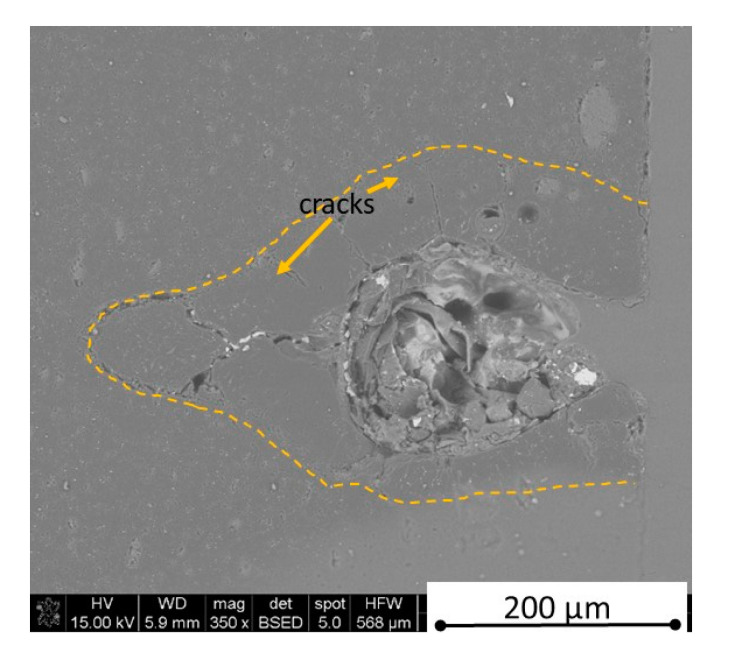
SEM observations of a 50 W laser processed B_4_C—8 vol.% h-BN in pressing direction (high surface roughness).

**Figure 31:**
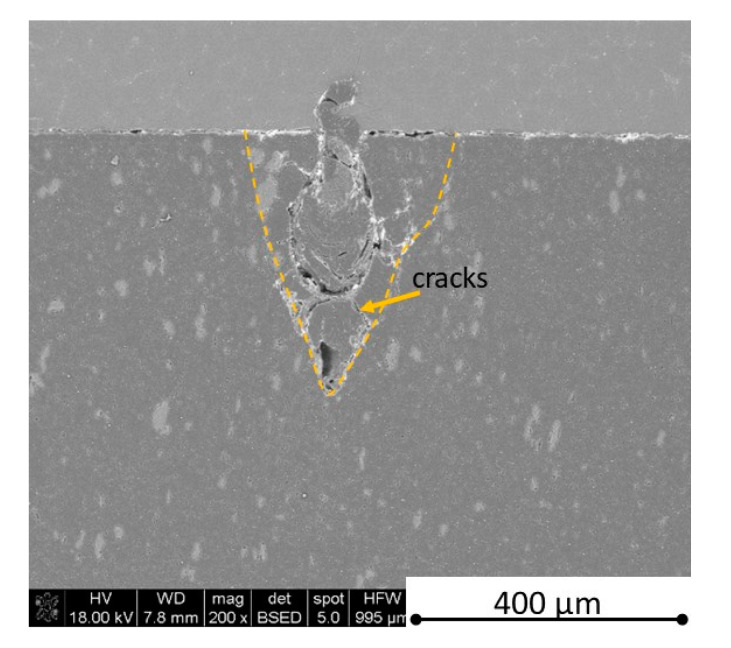
SEM observations of a 50 W laser processed B_4_C—8 vol.% h-BN in perpendicular direction (high surface roughness).

**Figure 32:**
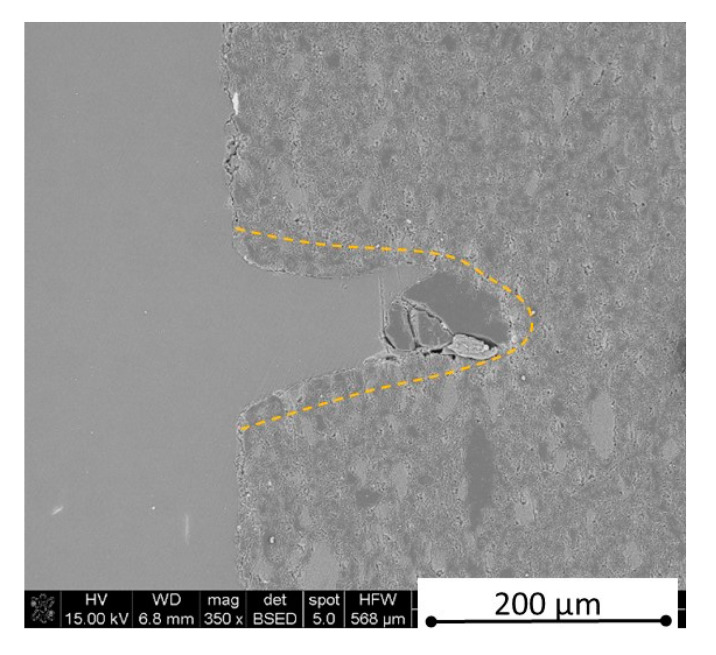
SEM observations of a 50 W laser processed B_4_C—32 vol.% h-BN in pressing direction (high surface roughness).

**Figure 33:**
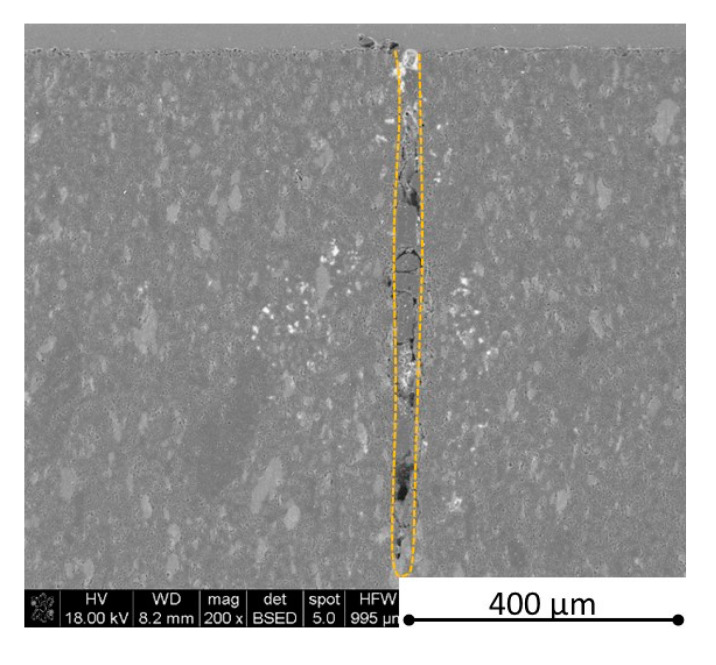
SEM observations of a 50 W laser processed B_4_C—32 vol.% h-BN in perpendicular direction (high surface roughness).

**Figure 34:**
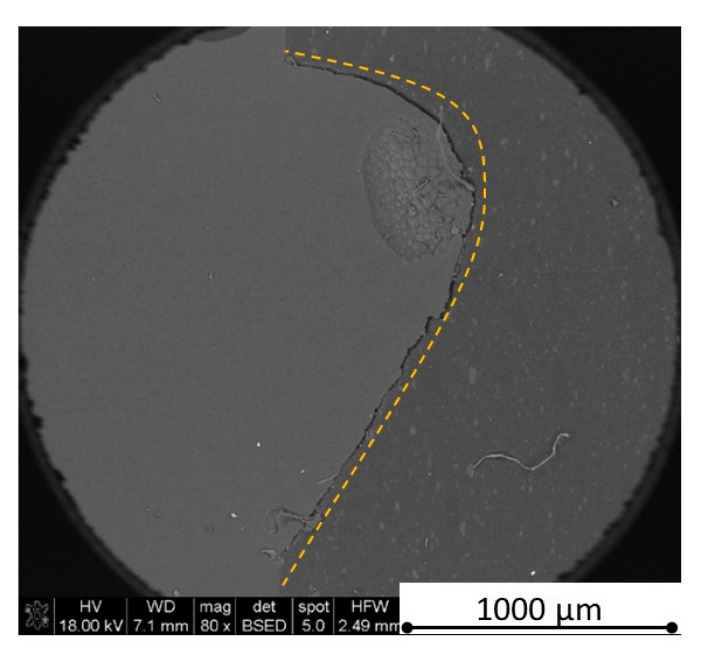
SEM observations of a 50 W laser processed B_4_C—8 vol.% h-BN in pressing direction (low surface roughness).

**Figure 35:**
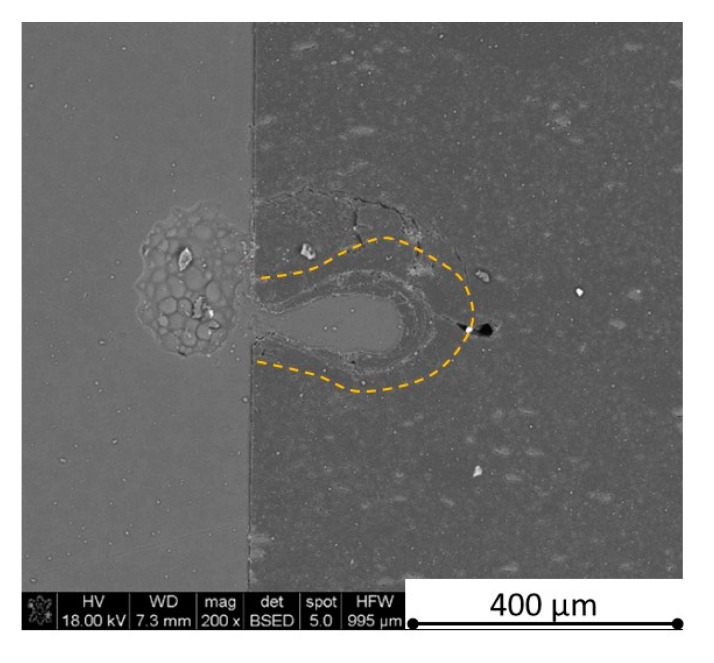
SEM observations of a 50 W laser processed B_4_C—8 vol.% h-BN in perpendicular direction (low surface roughness).

**Figure 36:**
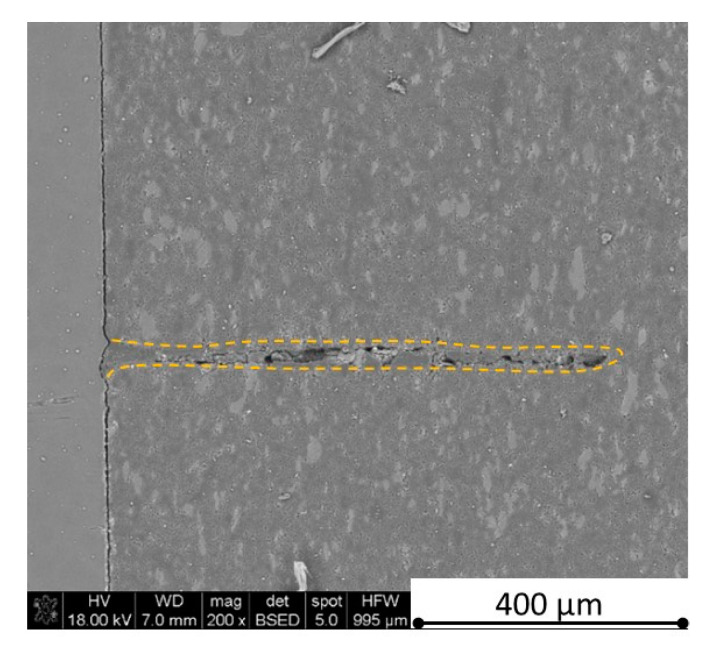
SEM observations of a 50 W laser processed B_4_C—32 vol.% h-BN in pressing direction (low surface roughness).

**Figure 37:**
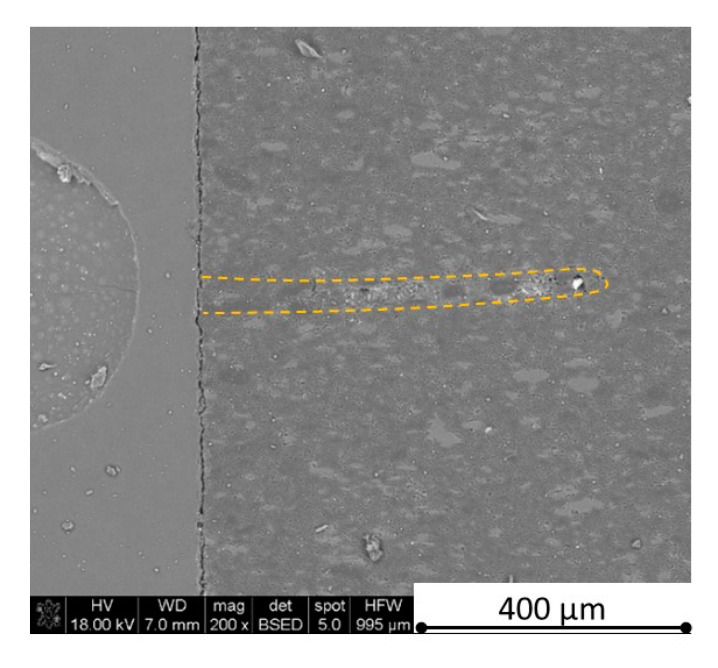
SEM observations of a 50 W laser processed B_4_C—32 vol.% h-BN in perpendicular direction (low surface roughness).

There is some information in the literature about ceramic laser machining mostly in a pulse mode and also in a continuous wave mode. The available example of an 8 mm thick ceramic tails laser cutting was shown by Black and Chua [26] in 1997. Their results confirmed that the pulse and continuous (CW) wave condition needs the multi-pass process, while in the case of CW, it was 60 passes, which is around 150 µm depth of a single cut. They were working with both the laser mode and CO_2_ laser. For alumina it is 200 to 400 µm, and for a good quality silicon carbide it is up to 300 µm in the laser pulse mode using the Yb:KGW laser (city, country) [27]. The laser ceramic treatment depth was show also in publication [28]. However, there is no information concerning the laser cutting of anisotropic ceramic composites. In this study, the laser treated materials were taken into the laser cutting process using a standard cutting laser head, argon flow, and continuous wave working mode. The process was done and analyzed in two different material directions and for the material roughness (R_a_) it was much lower and much higher than the wave length. For grinded materials with R_a_ around 2.69 µm, the observations are shown in Figure 26, Figure 27, Figure 28, Figure 29, Figure 30 and Figure 31 and for polished composites with R_a_ around 0.56 µm, the observations are presented in Figure 32, Figure 33, Figure 34, Figure 35, Figure 36 and Figure 37. A too low material roughness will lead to a light reflection. In this case, it gives a slightly better material treatment than in the case of a minimum of 2 times higher roughness towards the used laser wave length. In the case of a too high material (R_a_), some energy absorbed by surface unevenness tends to generate some heat that is not involved in the material processing. Therefore, a too rough surface makes the effect of laser processing slightly lower in the perpendicular material direction than in the case of polished surface for a high amount of h-BN. For low concentrations of introduced hexagonal boron nitrides, there are visible cracks (Figure 28, Figure 29, Figure 31 and Figure 35 ) or materials which are almost destroyed (Figure 27 and Figure 34). Lots of cracks appear in the remelted areas (Figure 28). Cracks and material destruction during laser processing can appear as an effect of a large CTE difference between boron carbide and hexagonal boron nitride single phases (Figure 14, Figure 15, Figure 16 and Figure 17). From the authors’ experience, similar cracks can possibly appear as an effect of h-BN decomposition and released nitrogen overpressure or in the case of residual water adsorbed in h-BN agglomerates.

The increase in h-BN content above 8 vol.% causes the change of the laser cut shape, which is presented in Figure 30 and Figure 31. For higher h-BN concentrations, the thermal anisotropy and h-BN agglomerates of the located porosity have a significant influence in laser processing. In perpendicular direction to the pressing axis (in-plane), the thermal diffusivity/conductivity is about 60% higher than in the pressing axis direction. It causes a heat transfer limitation in the out-of-plane direction and results in only 250–320 microns of processing depth for the rough surface sample. In this case, the ablation width is about 100 microns due to the heat transfer to the zone sides (Figure 32). There are no large cracks most likely due to the higher material porosity (Figure 3). Moreover, in the case of the low roughness polished sample with 32 vol.% h-BN, narrow, crack free, and deep laser cuts were observed (Figure 36 and Figure 37). Due to the argon flow some material stays inside the cut, but it can be removed by use of short pulse laser processing and a vacuum or under pressure conditions. In most of the laser treatment cases, the anisotropy of thermal properties leads to deeper and narrower cuts. For a low concentration of h-BN, cuts have the shape of a droplet.

The anisotropy of thermal properties, h-BN content, and material roughness also has an influence on the width end depth of the material cut. Comparing samples containing 0, 8, and 32 vol.% of h-BN in the case of the material with high surface roughness which is higher than laser wavelength, the cut depth changes in following way: in parallel direction to pressing axis: 320 µm for reference sample, 260 µm for 8 vol.% h-BN and 310 µm for 32 vol.%. Therefore, in this direction, the cut depth is typical as for the ceramic materials. It changes in perpendicular direction: 360 µm for reference, 400 µm for 8 vol.% h-BN, and 720 µm for 32 vol.% h-BN. The width of the cut in the case of pure boron carbide can reach 200 microns in the case of material destruction. For 8% h-BN, the width of the cut is around 100 microns in the pressing direction and 70 microns in the perpendicular direction. For 32%, the width of the cut is around 145 microns in the pressing direction and 40 microns in the perpendicular direction. By analyzing the dimension of the laser process ceramics with a surface roughness below the beam length, the reference sample shows the cut around 300 µm in both material directions. For the high polished surface in the case of 8 vol.% h-BN addition, the measured depth is around 230 µm and the width is 75 microns in perpendicular direction to the pressing axis, therefore, it is similar to the pressing direction. An interesting situation was noticed in the case of 32 vol.% of 2D particles. In this case, for the well-polished surface the depth is 150 µm higher than the perpendicular direction (610 µm). The difference is also visible in the width, where 60 µm is for the pressing direction and 40 µm for the perpendicular direction to the pressing axis. Here, it can be suggested that the reflectivity and energy absorption rate should also be studied in the future.

**Figure 38:**
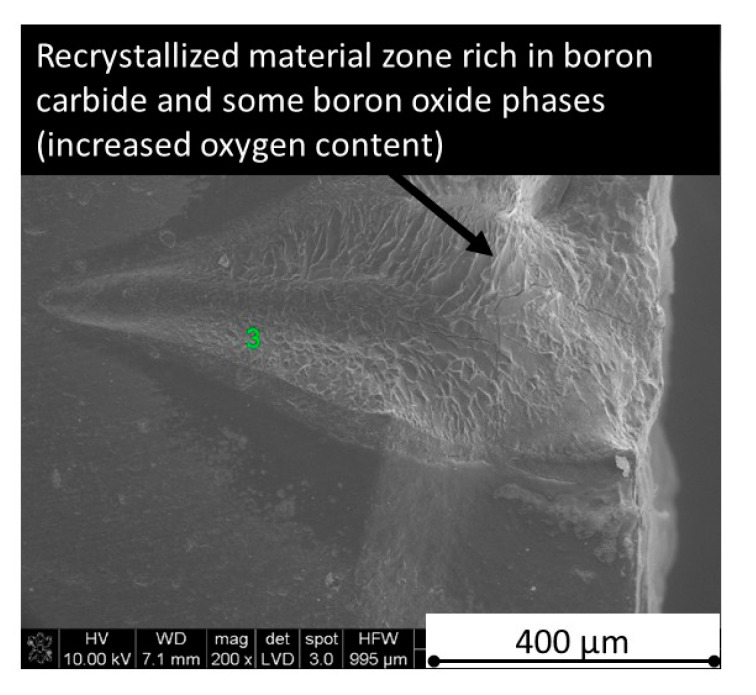
Cross-section of B_4_C—1% h-BN process with 90 W.

Concerning the chemical phenomena taking place during laser processing, not only the decomposition of h-BN (about 2500 °C) should be taken under attention, but also the melting point of pure stoichiometric boron carbide (2450 °C) and eutectics in the system B_4_C-C (2375 °C) and B_4_C-B (2075 °C) [29]. The confirmation of liquid phase and boron carbide formation in the cut tip is observed in Figure 32 and revealed by the EDS analysis showing only boron and carbon elements. The recrystallized phase is also visible in Figure 38.

## 4. Conclusions

During the research, anisotropic materials with a high relative density of over 94% were obtained by hot-pressing B_4_C with different amounts of h-BN addition. The laser processing conducted on all samples revealed a correlation between the h-BN content and shape of the performed cut. Additionally, the microstructure and thermal properties anisotropy is also dependent on the h-BN content in the samples. The porosity comes mostly from the h-BN agglomerates. A high anisotropy of the thermal expansion coefficient was observed between different material directions. It quickly reaches high values even for a low h-BN concentration, which can lead to material cracking during laser processing. The anisotropy of thermal conductivity and diffusivity was determined by LFA measurements. The thermal properties decrease in parallel direction to the pressing axis and slightly increase in perpendicular direction to the pressing axis. There is a high thermal properties anisotropy at low and high temperatures especially for the higher h-BN volume fraction. The observed high anisotropy of thermal properties influences the laser treatment of B_4_C/h-BN composites, resulting in deeper and narrower cuts after laser processing. The presence of a small amount of porosity in the material can protect composites from cracking during the laser action. The material surface roughness has little influence on laser processing and can be neglected. Future experiments employing the pulse working mode of laser and specific wave absorption coefficients are needed for a better understanding of the phenomenon during laser processing.

## Figures and Tables

**Table 1:** Phase composition of the B_4_C-h-BN mixture after the homogenization step.

Material	Phase Composition of Mixtures (wt %)				
	B_4_C-I	B_4_C-II	h-BN-I	h-BN-II	WC
B_4_C		0	0	0	
B_4_C/0.5 h-BN	47.0	50.7	1.1	1.1	0.1
B_4_C/1 h-BN	46.6	49.5	1.6	2.1	0.2
B_4_C/2 h-BN	45.1	50.3	2.0	2.4	0.2
B_4_C/4 h- BN	43.2	50.6	3.3	2.7	0.2
B_4_C/8 h-BN	46.5	44.9	5.2	3.2	0.2
B_4_C/16 h-BN	40.9	44.1	10.2	4.6	0.2
B_4_C/32 h-BN	34.1	42.0	4.4	19.4	0.1

**Table 2:** Mathematical function of density changes fitted to the experimental data (T-temperature (°C)).

vol.% of h-BN	Mathematical Function of Density Changes (g/cm^3^)	Fitting
0	y = −9 × 10^−9^T^2^ − 3 × 10^−5^T + 2.5216	R² = 0.9998
0.5	y = −1 × 10^−8^T^2^ − 3 × 10^−5^T + 2.5192	R² = 0.9996
1	y = −9 × 10^−9^T^2^ − 3 × 10^−5^T + 2.5109	R² = 0.9997
2	y = −1 × 10^−8^T^2^ − 3 × 10^−5^T + 2.5064	R² = 0.9993
4	y = −1 × 10^−8^T^2^ − 3 × 10^−5^T + 2.4891	R² = 0.9998
8	y = −1 × 10^−8^T^2^ − 3 × 10^−5^T + 2.4491	R² = 0.9996
16	y = −1 × 10^−8^T^2^ − 3 × 10^−5^T + 2.3812	R² = 0.9996
32	y = −8 × 10^−9^T^2^ − 3 × 10^−5^T + 2.3020	R² = 0.9999

**Table 3:** Mathematical function of heat transfer values changes fitted to the experimental data.

vol.% h-BN	Direction	Diffusivity mm^2^/s A = −Aln(T) + B		Conductivity W/(m K) λ = AT^3^ + BT^2^ + CT + D			
		A	B	A	B	C	D
0	Parallel	2.801	21.282	−1 × 10^−9^	2 × 10^−5^	−0.0351	31.093
2	Parallel	3.029	22.911	−5 × 10^−9^	3 × 10^−5^	−0.0417	33.295
	Perpendicular	3.149	23.780	−1 × 10^−9^	2 × 10^−5^	−0.0409	34.366
4	Parallel	2.950	22.372	−2 × 10^−9^	2 × 10^−5^	−0.0387	32.156
	Perpendicular	3.257	24.599	−4 × 10^−9^	3 × 10^−5^	−0.0449	35.327
8	Parallel	2.750	21.039	2 × 10^−10^	1 × 10^−5^	−0.0305	29.424
	Perpendicular	3.140	24.124	−5 × 10^−9^	3 × 10^−5^	−0.0397	34.282
16	Parallel	2.559	19.794	5 × 10^−9^	2 × 10^−6^	−0.0221	26.678
	Perpendicular	3.395	26.243	6 × 10^−9^	9 × 10^−6^	−0.0331	35.631
32	Parallel	2.066	16.418	1 × 10^−8^	−1 × 10^−5^	−0.0086	21.088
	Perpendicular	3.501	27.643	5 × 10^−8^	6 × 10^−5^	−0.0038	34.929
